# Quantitative myocardial blood flow imaging with integrated time-of-flight PET-MR

**DOI:** 10.1186/s40658-016-0171-2

**Published:** 2017-01-06

**Authors:** Tanja Kero, Jonny Nordström, Hendrik J. Harms, Jens Sörensen, Håkan Ahlström, Mark Lubberink

**Affiliations:** 1 Nuclear Medicine & PET, Uppsala University, Uppsala, Sweden; 2 Radiology, Department of Surgical Sciences, Uppsala University, Uppsala, Sweden; 3 Center for Research and Development, Uppsala/Gävleborg County Gävle, Sweden; 4 Department of Nuclear Medicine and PET, Århus University Hospitals, Århus, Denmark; 5 PET Center/Medical Imaging Center, Uppsala University Hospital, 75185 Uppsala, Sweden

**Keywords:** PET-MR, Myocardial blood flow (MBF), ^15^O-water, Quantification, Time-of-flight (TOF)

## Abstract

**Background:**

The use of integrated PET-MR offers new opportunities for comprehensive assessment of cardiac morphology and function. However, little is known on the quantitative accuracy of cardiac PET imaging with integrated time-of-flight PET-MR. The aim of the present work was to validate the GE Signa PET-MR scanner for quantitative cardiac PET perfusion imaging. Eleven patients (nine male; mean age 59 years; range 46–74 years) with known or suspected coronary artery disease underwent ^15^O-water PET scans at rest and during adenosine-induced hyperaemia on a GE Discovery ST PET-CT and a GE Signa PET-MR scanner. PET-MR images were reconstructed using settings recommended by the manufacturer, including time-of-flight (TOF). Data were analysed semi-automatically using Cardiac VUer software, resulting in both parametric myocardial blood flow (MBF) images and segment-based MBF values. Correlation and agreement between PET-CT-based and PET-MR-based MBF values for all three coronary artery territories were assessed using regression analysis and intra-class correlation coefficients (ICC). In addition to the cardiac PET-MR reconstruction protocol as recommended by the manufacturer, comparisons were made using a PET-CT resolution-matched reconstruction protocol both without and with TOF to assess the effect of time-of-flight and reconstruction parameters on quantitative MBF values.

**Results:**

Stress MBF data from one patient was excluded due to movement during the PET-CT scanning. Mean MBF values at rest and stress were (0.92 ± 0.12) and (2.74 ± 1.37) mL/g/min for PET-CT and (0.90 ± 0.23) and (2.65 ± 1.15) mL/g/min for PET-MR (*p* = 0.33 and *p* = 0.74). ICC between PET-CT-based and PET-MR-based regional MBF was 0.98. Image quality was improved with PET-MR as compared to PET-CT. ICC between PET-MR-based regional MBF with and without TOF and using different filter and reconstruction settings was 1.00.

**Conclusions:**

PET-MR-based MBF values correlated well with PET-CT-based MBF values and the parametric PET-MR images were excellent. TOF and reconstruction settings had little impact on MBF values.

## Background

Several imaging modalities are being used in assessment of myocardial perfusion and in detection of coronary artery disease (CAD). Unlike many other modalities and techniques, PET has the ability to measure myocardial blood flow (MBF) in absolute terms. The added value of quantitative MBF over qualitative myocardial perfusion imaging has been shown in several studies [1–5].

^15^O-water PET is considered to be the gold standard for non-invasive quantitative measurements of MBF [6, 7]. The short half-life of ^15^O (2 min) allows for measurement of both rest and hyperaemic MBF in less than half an hour. However, ^15^O-water is freely diffusible and is not, like other PET or SPECT perfusion tracers, trapped in the myocardium, thus making assessment of the left ventricular volumes and function technically very challenging. On the other hand, cardiac MRI has become the gold standard in assessment of myocardial volume, myocardial mass and ventricular function and is also used for tissue characterisation and vascular flow measurements.

Recently, integrated PET-MRI systems have become available, which allow for MBF measurements with PET and cardiac MRI simultaneously. Cardiac PET-MRI can give improved functional and morphological information (size, regional and global cardiac function, ejection fraction, stroke volume, intravascular flow measurements, tissue characterisation, etc.) compared to PET-CT or PET alone. Myocardial perfusion can be quantified with MRI, but it is technically demanding and although the coronary flow reserve (CFR) seems comparable between PET and MR, the absolute MBF values from PET and MR are only weakly correlated [8]. CFR is commonly used in the diagnosis of CAD; however, several studies have shown that absolute MBF at stress is superior to flow reserve in the detection of haemodynamically significant CAD [3, 9–11]. Combining MBF quantified with PET and functional and morphological information obtained with MRI is promising and will allow for a more comprehensive assessment in cardiac disease in a single patient visit. In addition, radiation doses can be reduced significantly because no CT is needed for attenuation correction of PET data.

However, dynamic scans with short-lived tracers such as ^15^O-water are among the biggest challenges to PET systems, because of the combination of very high count rates immediately after injection when all of the injected radioactivity is inside the field of view (FOV) of the scanner, and very low count rates at the end of the scan because of the near homogeneous distribution in the body and the passing of three radioactive half-lives. In addition, the larger axial FOV and smaller detector ring diameter compared to PET-CT result in a higher sensitivity, and hence higher count rates which presents a challenge for count rate linearity. These also result in a larger fraction of scattered radiation, which is further amplified by the presence of coils inside the FOV. Furthermore, attenuation correction based on MRI (MRAC) is still challenging [12, 13], and little is known on the quantitative accuracy of cardiac perfusion PET imaging with a PET-MR scanner. Hence, the performance of the PET systems in the new PET-MR scanners in relation to the measurement of MBF needs to be validated.

The aim of the present study was to validate a silicon photomultiplier (SiPM)-based time-of-flight (TOF) capable PET-MR scanner for quantitative cardiac PET imaging using ^15^O-water, by comparison to routine clinical PET-CT data. In addition to the cardiac PET-MR reconstruction protocol as recommended by the manufacturer, comparisons were made using a PET-CT resolution-matched reconstruction protocol both without and with TOF to assess the effect of time-of-flight and reconstruction parameters on quantitative MBF values.

## Methods

### Scanners

PET-CT scans were acquired on a Discovery ST PET-CT scanner (GE Healthcare, Waukesha). This scanner is equipped with 24 rings of 6 × 6 × 30 mm BGO detectors grouped in blocks of 6 × 6 crystals coupled to a single position-sensitive photomultiplier tube (PMT). The scanner produces 47 image slices with a slice thickness of 3.27 mm. The transaxial and axial FOV of the scanner are 70 and 15.7 cm, respectively. The system sensitivity according to the National Electrical Manufacturers Association NU-2 2007 standard is 9.1 cps/kBq [14].

PET-MR scans were acquired on a Signa PET-MR scanner (GE Healthcare, Waukesha). This scanner is equipped with 45 rings of 3.95 × 5.3 × 25 mm LYSO detectors grouped in blocks of 4 × 3 crystals coupled to 3 × 2 silicon photomultipliers (SiPM) each. SiPM gains are individually adjusted based on continuous temperature measurements to provide constant scanner sensitivity. The transaxial and axial PET FOV of the scanner are 60 and 25 cm, respectively. System sensitivity is 23 cps/kBq, and the scanner is capable of TOF-PET with a time resolution of circa 370 ps (manufacturer’s specifications and authors’ NEMA measurements).

### Phantom study

In order to establish which PET-MR reconstruction protocol resulted in images that were most comparable to our clinical routine PET-CT reconstructions, a NEMA image quality phantom with six fillable spheres (diameter 10, 13, 17, 22, 28 and 37 mm) was scanned in both scanners. The background of the phantom was filled with 20 MBq ^18^F and the spheres with a 10 times higher radioactivity concentration than the background, and the phantom was scanned on the Discovery PET-CT and the Signa PET-MR for 15 min each. PET-CT images were reconstructed using our clinical routine reconstruction parameters: ordered subsets expectation maximisation (OSEM) with 2 iterations, 21 subsets, and a 4.3 mm Gaussian post-filter. PET-CT attenuation correction was based on a low-dose CT scan. PET-MR images were reconstructed using OSEM with various numbers of iterations and subsets, different post filters, as well as without and with the use of TOF information. PET-MR attenuation correction was based on a built-in CT-based attenuation template of the phantom. One-centimeter diameter spherical volumes of interest (VOI) were automatically drawn over the centre of each sphere, and recovery for each sphere was calculated by dividing the measured radioactivity concentration to the known true radioactivity concentration. The PET-MR reconstruction method that best matched the PET-CT images was determined as the method with the smallest sum of squared residuals between PET-CT and PET-MR recovery coefficients for the three smallest spheres.

### Subjects

Eleven patients (nine male; mean age 59 years; range 46–74 years) participated in this prospective study. The patients had known or suspected CAD with intermediate pre-test probability of obstructive coronary disease (20–84% clinical pre-test probability) according to ESC Guidelines [15], and were referred for a ^15^O-water PET-CT study for evaluation of MBF. Written informed consent was obtained from all subjects, and the study was performed with permission from the local Radiation Ethics Committee and the Regional Board of Medical Ethics in Uppsala and in accordance with the declaration of Helsinki.

### Scan procedure

The subjects underwent ^15^O-water PET scans at rest and during adenosine-induced hyperaemia on both a GE Discovery ST PET-CT and a GE Signa PET-MR scanner on the same day (nine subjects) or within 4 days (two subjects). The radiation dose from the clinical PET-CT scan was approximately 1.8 mSv and the radiation dose from the PET-MR scan was approximately 0.8 mSv.

PET-CT: A 6-min dynamic PET perfusion scan during rest was started simultaneously with the administration of 400 MBq of ^15^O-water. After a 20–30-min delay to allow for decay of the remaining activity following the first injection, an identical PET scan was performed during adenosine-induced hyperaemia. Adenosine infusion 140 μg × kg^−1^ × min^−1^ was started 2 min prior to the stress scan and continued during the 6-min scan time. To correct for photon attenuation, a single low-dose respiration-averaged CT scan during normal breathing was acquired before the resting PET scan (140 kV, 10 mAs, rotation time 1 s, pitch 0.562). PET-CT images were reconstructed using OSEM (2 iterations, 21 subsets), applying all appropriate corrections such as for random coincidences, dead time, normalisation, and scatter, using a transaxial FOV of 50 cm and a 128 × 128 image matrix.

PET-MR: A 6-min dynamic PET perfusion scan during rest was started simultaneously with the administration of 400 MBq of ^15^O-water. After a 20–30-min delay following the first injection, an identical PET scan was performed during adenosine-induced hyperaemia as described above. Functional MR-imaging was obtained between the rest and stress PET-scans with a FIESTA (true FISP) cine sequence covering the left ventricular myocardium from apex to base in 8-mm-thick short-axis slices with 2.0 mm gap. To correct for photon attenuation, a two-point Dixon sequence during breath-hold was acquired during the resting PET scan and during the hyperaemic PET scan. This sequence enables segmentation of fat and water tissue, lungs and air, which form the basis for creation of the MR-based attenuation map. The arms, which are not included in the MR images, are added to the attenuation map from non-attenuation corrected TOF-PET data [16]. PET-MR images were reconstructed using OSEM into 128 × 128 pixel images and a FOV of 53.4 cm, using the cardiac protocol as recommended by the manufacturer (from here on referred to as std). To assess the effect of TOF and reconstruction settings on MBF values, PET-MR data were also reconstructed using the PET-CT resolution-matched protocol based on the phantom study, both without and with TOF. Reconstruction parameters are summarised in Table 1. All appropriate corrections such as for random coincidences, dead time, and normalisation were applied in all reconstructions.

**Table 1:** PET-MR reconstruction parameters

	Iterations	Subsets	Filter width (mm)	TOF
HD	2	28	6	No
FX	2	28	6	Yes
std	3	28	8	Yes

*TOF* time-of-flight

### Data analysis

The PET data was analysed semi-automatically using Cardiac VUer software, resulting in both parametric MBF images and segment-based MBF values for the entire left ventricle and for three regions corresponding to the coronary artery territories [17]. Coronary flow reserve (CFR) was defined as stress perfusion divided by rest perfusion and was calculated for each segment. The calculation of MBF was based on a one-tissue compartment model with an input function from arterial cluster analysis comprising left atrial and ventricular cavities and ascending aorta and with correction for spillover from left and right ventricular cavities into the myocardium:

1 $$ {C}_{\mathrm{PET}}(t)=\mathrm{P}\mathrm{T}\mathrm{F}\cdot \mathrm{M}\mathrm{B}\mathrm{F}\cdot {C}_A(t)\otimes {e}^{\frac{-\mathrm{M}\mathrm{B}\mathrm{F}}{V_T}t}+{V}_{\mathrm{LV}}{C}_A(t)+{V}_{\mathrm{RV}}{C}_{\mathrm{RV}}(t) $$

Here, *C*_PET_(*t*) is the radioactivity concentration as measured in a voxel or region by PET, PTF is the perfusable tissue fraction, *V*_*T*_ is the distribution volume of water, here fixed to 0.91 mL/g. *C*_*A*_(*t*) and *C*_RV_(*t*) are the radioactivity concentrations in arterial blood and in the right ventricular cavity, respectively, and *V*_LV_ and *V*_RV_ are the left- and right-ventricular spillover fractions. Parametric images were computed using a basis-function implementation of this model [17], whereas regional values were calculated using non-linear regression of Eq. 1. For PET-MR data, the cluster analysis and parametric image construction in Cardiac VUer were only performed for the standard clinical reconstruction protocol. For assessment of the regional MBF values for the other reconstruction methods, the blood vessel and regional myocardial VOIs resulting from the standard clinical analysis were projected onto the resolution-matched images both without and with TOF. Parametric MBF images from PET-CT and from PET-MR were compared visually.

To verify the count rate linearity of PET-MR during the first pass of the radioactivity through the PET FOV, the area under the time-activity curves from the arterial input functions during the first minute of the scans was compared for PET-CT and PET-MR. For this comparison, the arterial time-activity curves were normalised to their mean radioactivity concentrations during the last 4 min of the scan to account for possible small differences in amount of injected ^15^O-water.

The analysis of functional MR images was performed on a GE AW workstation using commercially available software (CardiacVX). The endocardial contour was semi-automatically traced and manually adjusted when needed. The ejection fraction was calculated with the software using Simpson’s rule.

### Statistical analyses

Continuous variables are presented as mean values ± standard deviation (SD). Comparison of the hemodynamic data, the global MBF and CFR values and the area under the time-activity curves from the input functions was performed by a Wilcoxon signed-rank test. Correlation and agreement between PET-CT and PET-MR-based regional MBF and CFR values were assessed using Deming regression and Bland-Altman analysis and intra-class correlation coefficients. A two-sided *p* value of less than 0.05 was considered significant. Statistical analyses were performed using SPSS (version 21.0).

## Results

**Fig. 1:**
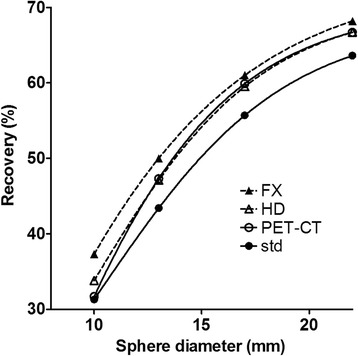
Recovery coefficients of the PET-CT images and of different PET-MR reconstructions. *Solid lines* are hyperbola fits to the measured data

Recovery coefficients of the PET-CT images reconstructed using our clinical routine protocol, as well as for a number of PET-MR reconstructions, are given in Fig. 1. Based on this data, a PET-MR reconstruction protocol without time of flight, using 2 iterations, 28 subsets and a 6 mm post-filter, resulted in recovery coefficients that were most similar to those for PET-CT.

Magnetic resonance imaging showed normal global systolic function in all subjects, with a mean ejection fraction (EF) of 65%; range 57–72%. MBF data from one patient was excluded because of movement during the PET-CT scan. Systolic blood pressure, heart rate and rate pressure product (RPP) were comparable between the PET-CT and the PET-MR scans as shown in Table 2.

**Table 2:** Hemodynamic and global MBF values

	PET-CT rest	PET-MR rest	*p*	PET-CT stress	PET-MR stress	*p*
SBP	135 ± 16	138 ± 17	ns	139 ± 14	133 ± 18	ns
HR	66 ± 8	65 ± 6	ns	86 ± 12	88 ± 10	ns
RPP	8970 ± 1665	8939 ± 1445	ns	11865 ± 2112	11728 ± 2326	ns
Global MBF	0.92 ± 0.12	0.90 ± 0.23	ns	2.74 ± 1.37	2.65 ± 1.15	ns
Global MBF corr	1.06 ± 0.27	1.02 ± 0.26	ns			

*SBP* systolic blood pressure (mmHg), *HR* heart rate (beats per minute), *RPP* rate pressure product (SBP × HR), *MBF* myocardial blood flow (mL/cm^3^/min), *MBF corr* myocardial blood flow corrected for RPP = (MBF/RPP) × 10^4^

**Fig. 2:**
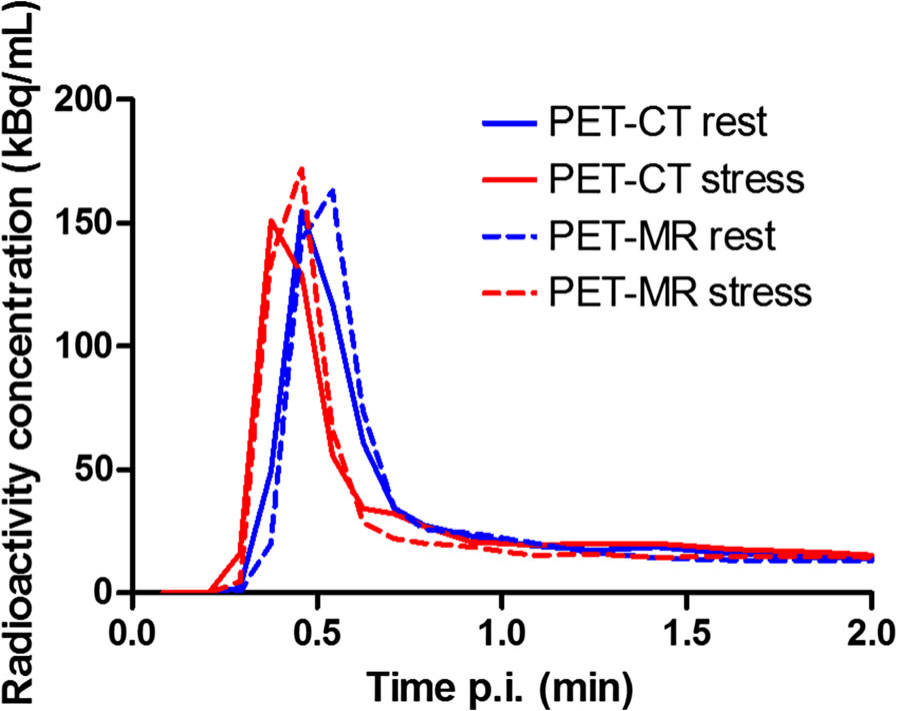
Time-activity curves of the arterial input function derived from cluster analysis comprising the left atrial and ventricular cavities and ascending aorta at rest and at stress in PET-CT and in PET-MR in a typical patient

An example of typical time-activity curves of the arterial input function at rest and at stress in PET-CT and in PET-MR is shown in Fig. 2. The mean area under the curves during the first 1 min for all patients (±SD) was 49.5 ± 9.6 kBq/ml × min for PET-CT and 48.0 ± 8.3 kBq/ml × min for PET-MR (*p* = 0.12).

**Fig. 3:**
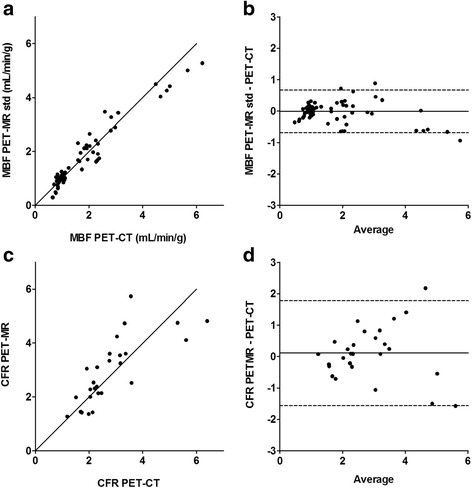
Correlation (**a**, **c**) and Bland-Altman plots (**b**, **d**) of PET-MR-based regional MBF std (clinical protocol) versus PET-CT-based regional MBF (**a**, **b**) and regional CFR (**c**, **d**). Rest and hyperemic stress values are plotted for MBF. The *solid lines* in **a** and **c** are lines of identity. The *solid lines* in **b** and **d** indicate the mean difference (bias), whereas the *dashed lines* show the limits of agreement. The regression slopes are 0.91 (0.85–0.98) and 0.98 (0.64–1.32) in **a** and **c**, respectively. Bias (limits of agreement) are −0.04 (−0.73 to 0.65) and 0.11 (−1.56 to 1.78) in **b** and **d**, respectively

**Fig. 4:**
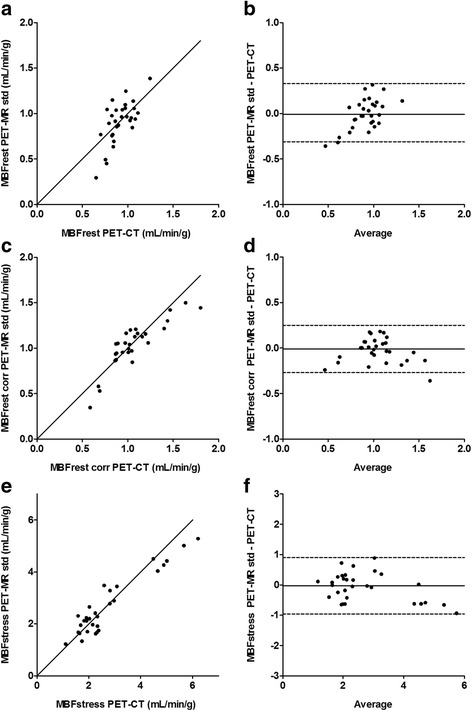
Correlation (**a**, **c** and **e**) and Bland-Altman plots (**b**, **d** and **f**) of PET-MR-based regional MBF std (clinical protocol) versus PET-CT-based regional MBF at rest (**a**, **b**), regional MBF at rest corrected for RPP (**c**, **d**) and at regional MBF at adenosine-stress (**e**, **f**). The *solid lines* in **a**, **c** and **e** are lines of identity. The *solid lines* in **b**, **d** and **f** indicate the mean difference (bias), whereas the *dashed lines* show the limits of agreement. Regression slopes are 2.12 (1.26–2.99), 0.92 (0.72–1.11) and 0.85 (0.73–0.97) in **a**, **c** and **e**, respectively. Bias (limits of agreement) are −0.008 (−0.34 to 0.33), −0.03 (−0.29 to 0.23) and −0.07 (−0.99 to 0.85) in **b**, **d** and **f**, respectively

**Fig. 5:**
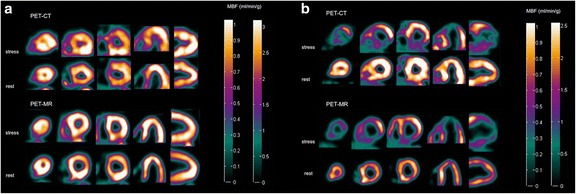
Parametric images of stress and rest MBF from PET-CT and PET-MR std (clinical protocol) with Cardiac VUer software. Images from a patient with normal myocardial perfusion (**a**) and from a patient with apical, anterior and inferior ischemia (**b**). The colour scale is absolute (i.e. in mL/g/min) and identical for the PET-CT and the PET-MR images for each patient

Global mean (±SD) MBF values at rest and stress were 0.92 ± 0.12 and 2.74 ± 1.37 mL/g/min for PET-CT and 0.90 ± 0.23 and 2.65 ± 1.15 mL/g/min for PET-MR, respectively (*p* = 0.33 and *p* = 0.74). Global mean (±SD) CFR values were 2.97 ± 1.31 for PET-CT and 3.05 ± 1.23 for PET-MR (*p* = 0.65). The relations between PET-MR-based and PET-CT-based regional MBF and CFR are shown in Fig. 3. Intra-class correlation coefficients (ICC) between PET-CT and PET-MR regional MBF and CFR were 0.98 and 0.89, respectively. The agreement between PET-MR-based and PET-CT-based regional MBF at rest, at rest corrected for rate-pressure-product (RPP) and at stress is shown in Fig. 4. Intra-class correlation coefficients (ICC) between PET-CT-based and PET-MR-based regional MBF at rest, corrected rest and stress were 0.76, 0.93 and 0.96, respectively. The image quality of parametric MBF images, as shown in Fig. 5, was excellent for PET-MR and in most cases, superior to the PET-CT images, based on visual assessment.

**Fig. 6:**
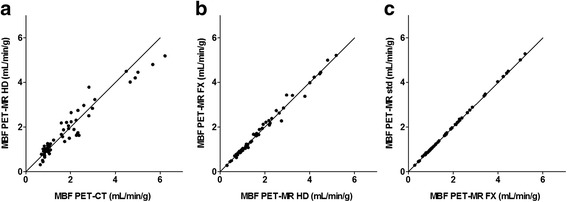
Resolution-matched PET-MR-based regional MBF without TOF (HD) versus PET-CT-based regional MBF (**a**), resolution-matched PET-MR-based regional MBF without TOF (HD) versus resolution-matched PET-MR-based regional MBF with TOF (FX) (**b**), and PET-MR FX versus clinical protocol (std) (**c**). *Solid lines* are lines of identity. Regression slopes are 0.90 (0.83–0.96), 0.98 (0.96–1.02) and 1.01 (1.00–1.01) in **a**, **b** and **c**, respectively

The agreement between resolution-matched PET-MR-based regional MBF with and without TOF (FX and HD) and with standard reconstruction (PET-MR std) is shown in Fig. 6. The ICC was 1.00 both between the PET-MR MBF with and without TOF and between PET-MR MBF std and FX-reconstruction.

## Discussion

This present study assessed the quantitative accuracy of cardiac perfusion measurements with ^15^O-water in the Signa PET-MR scanner. A high correlation and agreement between PET-MR-based and PET-CT-based MBF was found. This enables the application of previously established cut-off values for MBF with ^15^O-water PET-CT also in PET-MR studies [10].

In ^15^O-water cardiac scans, the count rates are very high in early time frames, which presents a challenge for count rate linearity of the PET scanner and reliable arterial input function definition, which is essential for the calculation of MBF. We recently performed a NEMA count rate linearity test of the PET-MR scanner as part of the scanner’s acceptance procedure, starting at a total amount of radioactivity of 950 MBq. This corresponds to an approximately 40 kBq/ml or 340 MBq in the field of view of the scanner, which is similar to the maximum amount encountered during the ^15^O-water scans if all the activity would be within the field of view during the first pass of the tracer. The measured radioactivity concentration did not deviate more than 5% from the true radioactivity concentration at any time during this test, so we are confident that the scanner behaves linearly during the scans and the arterial input function is recovered well. This was further verified by comparing the area under the arterial input function during the first 1 min for PET-CT and PET-MR scans, which did not differ significantly.

The small differences between the MBF measurements in the PET-CT and in the PET-MR can likely be attributed to physiologic variations of myocardial blood flow and lie well within the variability of repeated measurements of ^15^O-water myocardial perfusion at rest and during adenosine hyperaemia as reported by Kaufman et al. [18]. The repeatability coefficients for MBF (calculated as 1.96 × SD of the differences) they reported were 0.17, 0.28 and 0.90 for global rest, global corrected rest and global adenosine stress, respectively, and the repeatability coefficients for regional MBF were 0.20–0.46 at rest and 0.41–0.59 at adenosine stress. The repeatability coefficients for MBF measured in the PET-CT and in the PET-MR in our study, as shown in Table 3, are comparable to those reported by Kaufman et al.

**Table 3:** MBF and repeatability coefficients

	MBF PET-CT rest	MBF PET-MR rest	Repeatability coefficient		MBF PET-CT stress	MBF PET-MR stress	Repeatability coefficient	
			Absolute % of mean				Absolute % of mean	
Global	0.92 ± 0.12	0.90 ± 0.23	0.34	37%	2.74 ± 1.37	2.65 ± 1.15	0.84	31%
Global corrected	1.06 ± 0.27	1.02 ± 0.26	0.20	19%				
Regional	0.91 ± 0.14	0.91 ± 0.23	0.33	36%	2.74 ± 1.33	2.67 ± 1.14	0.92	34%

Repeatability coefficient = 1.96 × SD of differences

Although the agreement between PET-CT-based and PET-MR-based MBF was high, the small differences in MBF values could still result in different clinical decisions for the PET-CT and for the PET-MR-based studies. Using the previously established cut-off value of 2.3 mL/g/min to decide between normal and pathological stress MBF [10], on a subject-based level, five subjects had pathological MBF (at least one segment with MBF <2.3 mL/g/min) in the PET-CT study and all of these subjects also had pathological MBF in the PET-MR-based analysis. Five subjects had normal MBF in all segments in the PET-CT study; four of these subjects also had normal MBF in all segments in the PET-MR study whereas one subject had reduced MBF in all the segments (global MBF was 2.3 mL/g/min with PET-CT and 1.8 mL/g/min with PET-MR). This subject was one of the two subjects that underwent the PET-MR scan on a different day than the PET-CT scan; 3 days later. On a segment-based level 13 out of 30 segments had pathologically reduced MBF in the PET-CT study; 11 of these segments were also pathological with the PET-MR-based analysis whereas 2 were normal. Seventeen segments had normal MBF in the PET-CT study and 13 of these regions were also normal with the PET-MR-based analysis, whereas 4 segments were pathological. Altogether, in 24 out of 30 segments, the PET-CT and the PET-MR-based decisions of normal or reduced MBF agreed and in 6 segments, they did not agree. Four of these six segments that did not agree were in the two patients that underwent the PET-CT and PET-MR studies on different days. Patients were requested to not alter any medications between the PET-CT and the PET-MR scans and to withhold from caffeine during 24 h before both PET-scans, but failure in compliance to this or other physiologic reasons, rather than differences in the scanners, may have influenced the results and we feel confident in trusting the clinical decisions based on the MBF values using the GE Signa PET-MR scanner. Considering the similar ICC values of the present PET-CT – PET-MR comparison and the variability study by Kauffman et al., it is likely that similar differences in clinical diagnoses would have occurred if PET-CT scans had been repeated. When using a fixed cut-off value for pathological MBF, there is always a probability that patients with MBF close to this cut-off value will be diagnosed differently based on different scans even with the relatively high reproducibility of MBF measurements.

CFR is commonly used in the diagnosis of CAD, although several studies have shown that absolute MBF at stress is superior to flow reserve [3, 9–11]. In our study, MBF values showed better agreement between PET-CT and PET-MR than CFR values, as shown in Fig. 2.

As shown in Fig. 6a, the relation between the resolution-matched PET-MR data and the PET-CT data was virtually identical to the relation between the clinical standard PET-MR data and PET-CT data depicted in Fig. 3. Indeed, as Fig. 6b, c shows, the standard cardiac PET-MR reconstruction applying 3 iterations, 28 subsets and an 8 mm post-filter produced nearly identical MBF values to the PET-CT-resolution-matched reconstruction with 2 iterations, 28 subsets and a 6 mm filter, both without (HD) and with (FX) TOF. Although this may seem counterintuitive, this is probably due to the fact that for ^15^O-water, MBF is based on the clearance rate, i.e., the exponential term in Eq. 1, of the tracer instead of the amplitude of the myocardial time-activity curve (TAC). Additional filtering does affect this amplitude, but not the shape of the myocardial TAC, and hence does not affect the clearance rate. This means that for ^15^O-water, additional filtering does not decrease MBF, whereas it would for other flow tracers.

In the PET-MR, the MRAC is still a matter of concern; the attenuation map does not differentiate between the soft tissue and bone, which can result in underestimation of the PET signal [19, 20]. A recent study showed comparable relative myocardial FDG uptake in PET-MR and PET-CT images [16] but little is known on the impact of attenuation on the quantitative accuracy of cardiac perfusion in the PET-MR. For PET-CT, it has been shown that MBF can be measured accurately with ^15^O-water without correcting for attenuation [21]. Errors in attenuation correction affect the amplitudes of the time-activity curves but not their shapes, and hence not the measured clearance rates. Indeed, in our study, a high agreement was found between PET-MR-based and PET-CT-based MBF, suggesting that the potential errors in MRAC have little impact on the ^15^O-water MBF values. This result cannot readily be extrapolated to other tracer used for measuring MBF such as ^82^Rb and ^13^N-ammonia, since for those tracers MBF is determined from the uptake instead of the clearance of the tracer.

TOF-PET imaging is an emerging imaging technology both for PET-CT and PET-MR. In a recent study, Mehranian et al. assessed the impact of TOF image reconstruction on PET quantification errors induced by MR-based attenuation correction in ^18^F-FDG and ^18^F-choline whole body PET-MR scans [22]. They showed that TOF substantially reduced artefacts and significantly improved the quantitative accuracy. In recent cardiac PET-CT studies with ^13^N-ammonia and ^82^Rubidium, TOF reconstruction also improved image quality and increased MBF [23, 24]. In our study, we did not find any significant impact of TOF and filter and reconstruction settings on the quantitative accuracy of cardiac perfusion measurements with ^15^O-water in the PET-MR. However, the parametric PET-MR MBF images with TOF were excellent and in most cases, the image quality was visually superior to the PET-CT images. We did not evaluate the effect of TOF, filter and reconstruction settings on parametric image quality, as well as in PET-CT, [24, 25] TOF is expected to improve image quality and to make it possible to find smaller perfusion defects, which should be evaluated in a further study.

The results of this study are depending on the specific technology of the Signa SiPM PET-MRI scanner and reconstruction methods used. Quantitative accuracy of MBF values obtained using other PET-MR scanners and tracers should be validated in a similar manner. However, with ^15^O-water offering the largest challenge to PET scans in terms of count rate variations possibly with the exception of ^82^Rb, we expect that the results in the present work in terms of PET performance are also valid for dynamic myocardial imaging with other tracers.

## Conclusions

Cardiac perfusion measurements with ^15^O-water can be performed accurately with the fully integrated Signa PET-MR scanner. MR-based attenuation correction, TOF and reconstruction settings have little impact on the quantitative MBF values. Cardiac PET-MRI allows for quantitative assessment of MBF combined with the superior functional and morphological information from MRI.
